# 7,8,4′-Trihydroxyisoflavone Attenuates DNCB-Induced Atopic Dermatitis-Like Symptoms in NC/Nga Mice

**DOI:** 10.1371/journal.pone.0104938

**Published:** 2014-08-29

**Authors:** Heejung Kim, Jong Rhan Kim, Heerim Kang, Jinhwan Choi, Hee Yang, Pomjoo Lee, Jiyoung Kim, Ki Won Lee

**Affiliations:** 1 WCU Biomodulation Major, Department of Agricultural Biotechnology, Seoul National University, Seoul, Republic of Korea; 2 Advanced Institutes of Convergence Technology, Seoul National University, Suwon, Republic of Korea; 3 Center for Food and Bioconvergence, Seoul National University, Seoul, Republic of Korea; 4 Laboratory of Toxicology, Research Institute for Veterinary Science, College of Veterinary Medicine, Seoul National University, Seoul, Republic of Korea; 5 Research Institute of Bio Food Industry, Institute of Green Bio Science and Technology, Seoul National University, Pyeongchang, Republic of Korea; CNRS-University of Toulouse, France

## Abstract

Atopic dermatitis (AD) is characterized by chronic highly pruritic and relapsing inflammatory skin lesions. Despite its growing prevalence, therapeutic treatments remain limited. Natural immune modulators from herbal extracts or derivatives may be useful for treating AD symptoms. This study examined the effect of 7,8,4**′**-trihydroxyisoflavone (7,8,4**′**-THIF), a metabolite of soy isoflavone daidzin, on AD-like symptoms. Repeated epicutaneous application of 2,4-dinitrochlorobenzene (DNCB) was performed on the ear and dorsal skin of NC/Nga mice to induce AD-like symptoms and skin lesions, and 7,8,4**′**-THIF (200 and 400 nmol) or tacrolimus (100 µg) was applied topically for 3 weeks to assess their anti-pruritic effects. We found that 7,8,4**′**-THIF alleviated DNCB-induced AD-like symptoms as quantified by skin lesion, dermatitis score, ear thickness, and scratching behavior. Histopathological analysis demonstrated that 7,8,4**′**-THIF decreased DNCB-induced eosinophil and mast cell infiltration into skin lesions. We also found that 7,8,4**′**-THIF significantly alleviated DNCB-induced loss of water through the epidermal layer. In addition to reducing the DNCB-induced increase in serum IgE, 7,8,4**′**-THIF also lowered skin lesion levels of the chemokine thymus and activation regulated chemokine; Th2 cytokines interleukin (IL)-4, IL-5, and IL-13; and Th1 cytokines IL-12 and interferon-γ. These results suggest that 7,8,4′-THIF might be a potential therapeutic candidate for the treatment of atopic dermatitis.

## Introduction

Atopic dermatitis (AD) is a chronic inflammatory skin disease that is increasingly more common in infants and children [1], [2]. The clinical symptoms of AD are characterized by elevated serum immunoglobulin E (IgE) levels and pruritic and relapsing eczematous skin lesions, which are distinguished by epidermal thickening; defective skin barriers; and infiltration of inflammatory cells, such as lymphocytes, macrophages, eosinophils, and mast cells [1]–[5]. T-helper 2 (Th2) cells producing thymus and activation-regulated chemokine (TARC), interleukin (IL)-4, IL-5, and IL-13 play major roles in AD onset and development [6]–[9]. Although Th2 cells are dominant during the acute phase of AD, IL-12- and interferon (IFN)-γ-producing Th1 cells are highly expressed and contribute to the pathogenesis during the chronic phase [9]–[11]. For many years, AD therapeutic strategies have been dominated by the application of local or systemic corticosteroids [12]. However, these steroids often produce adverse effects in patients with AD [13], and there is a great need to develop new and effective AD therapies.

The NC/Nga mouse is the first reported spontaneously occurring AD model [14], [15]. Skin changes that closely mimic human AD are induced in NC/Nga mice following exposure to various environmental aeroallergens [14]. It was reported that 2,4-dinitrochlorobenzene (DNCB), an electrophilic and cytotoxic benzene derivative, induces stable clinical AD-like skin diseases in NC/Nga mice [16]–[18]. Skin changes in NC/Nga mice are evidenced by scratching behavior, followed by the rapid development of erythema, lichenification with edema, and hemorrhage [14], [19]. Histological examinations have revealed hyperplasia and dense accumulation of eosinophils and mast cells in skin lesions [14], [17], [20]. Along with these skin changes, NC/Nga mice exhibit elevated levels of total serum IgE [14], [15]. DNCB-induced contact hypersensitivity pathogenesis is predominantly the result of T cell-mediated immune responses [21].

Daidzin and genistin are naturally occurring isoflavones that are highly concentrated in soybeans [22]. Genistein, a metabolite of genistin, has been shown to exert beneficial effects in animal models of arthritis, asthma, and spontaneous severe dermatitis [23]–[25], whereas daidzin and its metabolites have been studied less. 7,8,4′-Trihydroxyisoflavone (7,8,4′-THIF, 8-hydroxydaidzein), a metabolite of daidzin, has recently attracted much attention in view of its pharmaceutical and cosmetic effects [26]. Here, we investigated whether topical application of 7,8,4′-THIF suppressed the development of AD-like symptoms and skin lesions in DNCB-treated NC/Nga mice by quantifying dermatitis score, ear thickness, scratching behavior, and serum IgE production. The effects of 7,8,4′-THIF on eosinophil and mast cell infiltration and transepidermal water loss (TEWL) were investigated in skin lesions. The effects of 7,8,4′-THIF on TARC and Th2 and Th1 cytokine levels in skin lesions were also investigated. The effects of 7,8,4′-THIF were compared with those of tacrolimus, an immunosuppressant commonly used to treat AD (eczema).

## Materials and Methods

### Ethics Statement

All animal procedures were performed in accordance with the Guide for the Care and Use of Laboratory Animals (Washington DC, USA) and were approved by the Institutional Animal Care and Use Committee of Seoul National University, Seoul, Korea (SNU-110211-2).

### Animals and treatment

**Figure 1:**
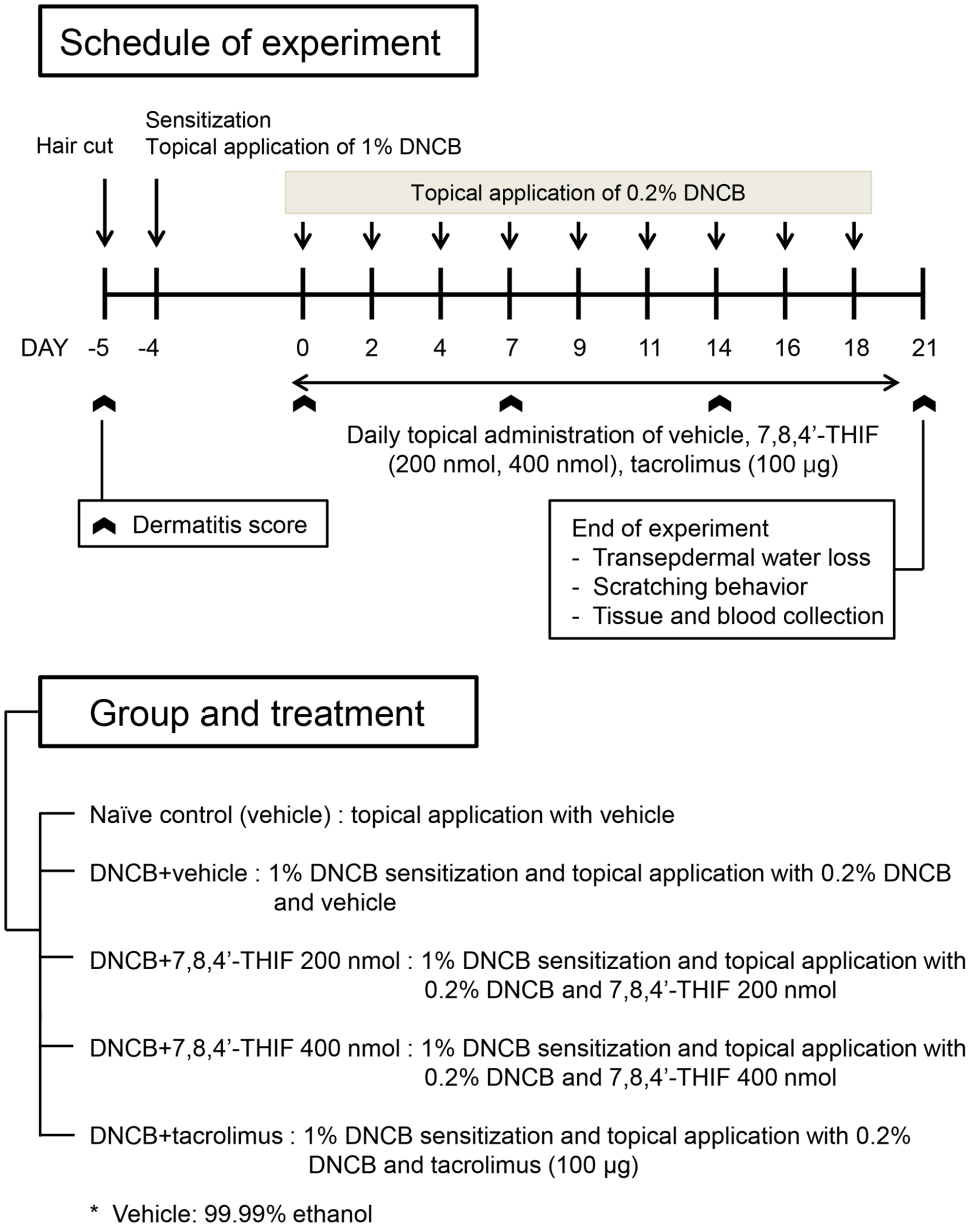
Experimental design. NC/Nga mice were divided into five groups: (1) naïve control (vehicle), (2) DNCB+vehicle, (3) DNCB+200 nmol 7,8,4′-THIF, (4) DNCB+400 nmol 7,8,4′-THIF, and (5) DNCB+tacrolimus. To induce AD-like symptoms, DNCB was topically applied on NC/Nga mice. A day after complete dorsal hair removal, 1% DNCB was applied (day −4). Five days after dorsal hair removal, 0.2% DNCB was challenged three times a week for three weeks (day 0–20). 7,8,4′-THIF or tacrolimus was topically applied seven times a week for 3 weeks (day 0–20).

Four-week-old NC/Nga female mice were purchased from SLC Japan (Tokyo, Japan). Mice were housed in individual ventilated cages under specific pathogen-free conditions at 22±2°C with a 12-h light-dark cycle. After 2 weeks of acclimation, mice were divided into five groups (n = 9 per group): (1) naïve control (vehicle), (2) DNCB+vehicle, (3) DNCB+200 nmol 7,8,4′-THIF, (4) DNCB+400 nmol 7,8,4′-THIF, and (5) DNCB+tacrolimus. To induce AD-like symptoms and skin lesions, DNCB was applied to the dorsal skin, face, and the back of both ears of NC/Nga mice. A day after complete dorsal hair removal (approximately 4 cm^2^), 150 µl 1% DNCB dissolved in an acetone∶olive oil mixture (3∶1 vol/vol) was applied on the dorsal skin, and 10 µl each were applied to the face and the back of both ears (day −4). Five days after dorsal hair removal, 0.2% DNCB dissolved in an acetone∶olive oil mixture (3∶1 vol/vol) was applied to challenge the dorsal skin (150 µl) and the face and the back of both ears (10 µl each) three times a week for 3 weeks (day 0–20). 7,8,4′-THIF dissolved in ethanol (200 and 400 nmol/mouse/day, 150 µl/dorsal skin/day, 10 µl/face or back of both ears/day) or tacrolimus (0.1% Protopic Ointment, Astellas Pharma, Tokyo, Japan; 100 µg/mouse/day) was topically applied to the dorsal skin, face, and back of both ears, seven times a week for 3 weeks (day 0–20). At the end of experiments, animals were anesthetized with 2% isoflurane. The experimental design is summarized in Fig. 1.

### Skin lesion, dermatitis score, and ear thickness

Mice were anesthetized with 2% isoflurane, and images of skin lesions were taken using a digital camera (Canon SX40 HS, Tokyo, Japan) on the last day of the experiment (day 21). The dermatitis score was measured once a week according to a slight modification of the criteria described previously [27]. Scores of 0 (none), 1 (mild), 2 (moderate), and 3 (severe) were given for each of the four symptoms: (i) erythema/hemorrhage, (ii) edema, (iii) excoriation/erosion, and (iv) scaling/dryness. A total dermatitis score indicating clinical severity was defined as the sum of all scores (maximum score: 15). Mouse ear thickness was also measured and recorded once a week using a vernier caliper (Mitutoyo, Kanagawa, Japan).

### Scratching behavior

To investigate AD-like behavioral changes, we measured and recorded the time that NC/Nga mice spent rubbing their nose, ears, and dorsal skin with their hind paws for 20 min on the last day of the experiment (day 21).

### Histological examination

To evaluate epidermal thickening, the ear skin of each mouse was prepared on the last day of the experiment (day 21), fixed with 10% neutral-buffered formalin, and embedded in paraffin. Then, 4-µm-thick sections were cut and transferred onto slides. Deparaffinized skin sections were stained with hematoxylin and eosin (H&E) before they were examined at 100× magnification. To detect eosinophil and mast cell infiltration, the dorsal skin of each mouse was also prepared on the last day of the experiment (day 21) as described above. Deparaffinized skin sections were stained with Congo red (CR) and toluidine blue (TB), respectively. The number of eosinophils and mast cells per 0.025 µm^2^ skin was counted at 400× magnification. Tissue sections were examined using an Olympus AX70 light microscope (Tokyo, Japan).

### IgE, TARC, Th2, and Th1 cytokines

Blood and dorsal skin were collected on the last day of the experiment (day 21) and stored at −80°C until use. Serum IgE levels were measured using an enzyme-linked immunosorbent assay (ELISA) kit (Shibayagi, Gunma, Japan) according to the manufacturer's instructions. The dorsal skin levels of chemokine TARC; Th2 cytokines IL-4, IL-5, and IL-13; and Th1 cytokines IL-12 and IFN-γ were also measured by ELISA kits (R&D Systems, Minneapolis, MN, USA). Briefly, the tissue was homogenized in lysis buffer, and then the freezing/thawing procedure was repeated three times. After centrifugation, the supernatants containing total cellular protein were quantified and used to detect the level of chemokine and cytokines. Results were normalized to the total amount of protein prepared from tissue lysates.

### Transepidermal Water Loss

TEWL measures the quantity of water that passes from inside an animal's body to the surrounding atmosphere through the epidermal layer (skin) via diffusion and evaporation processes, and it was measured on the last day of the experiment (day 21). TEWL in mouse dorsal skin was measured under specific conditions at 21–22°C and 50–55% humidity, using a skin evaporative water recorder Tewameter TM300 (Courage and Khazaka, Cologne, Germany). Measurements were recorded when TEWL readings stabilized approximately 30 sec after the probe was placed on the skin. The data were analyzed with a microprocessor and are expressed in g/m^2^/h.

### Statistical analysis

Statistical analysis was performed with SPSS Statistics Software (SPSS Inc., Chicago, IL, USA). The data were expressed as means ± standard error of the mean (SEM). One-way analysis of variance (ANOVA) followed by Tukey's honestly significant difference (HSD) test was used for comparing differences among multiple groups. Differences were considered significant at *p*<0.05.

## Results

### 7,8,4′-THIF alleviated DNCB-induced AD-like symptoms in NC/Nga mice

**Figure 2:**
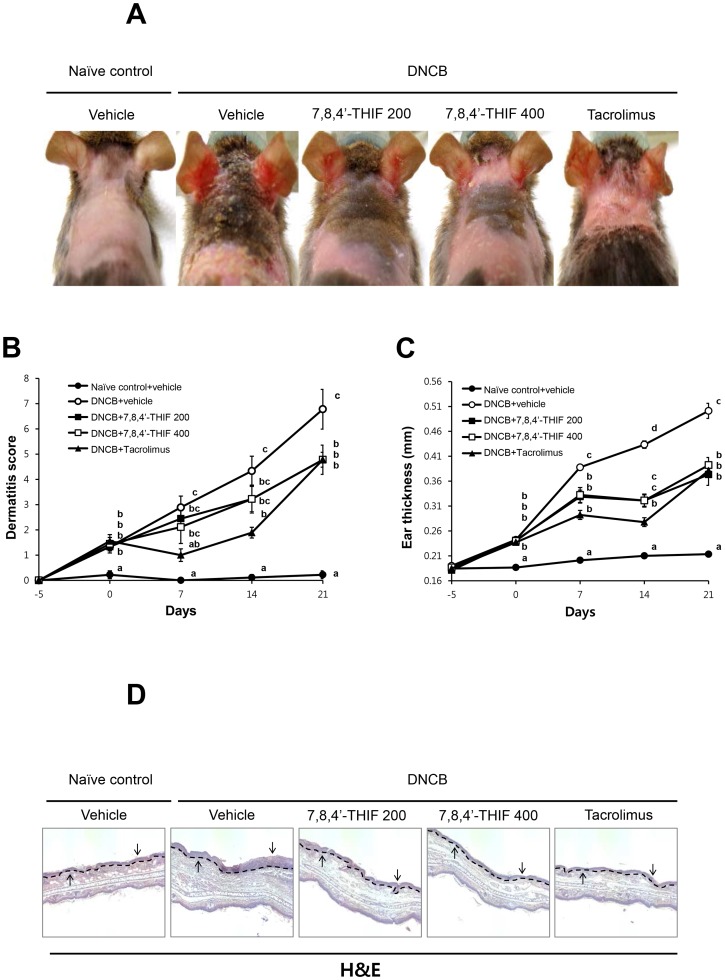
Effect of 7,8,4′-THIF on DNCB-induced AD-like symptoms in NC/Nga mice. (A) Images of skin lesions from the groups of mice were taken on the last day of the experiment (day 21). (B) Dermatitis scores were evaluated weekly from day −5 to 21. (C) Ear thickness was measured from day −5 to 21. (D) H&E-stained ear skin on day 21 (100×). Arrows indicate epidermal hypertrophy. Data represent the mean ± SEM (n = 9). Means with letters (a–d) within a graph are significantly different from each other at *p*<0.05.

To investigate the effects of 7,8,4′-THIF on DNCB-induced AD-like symptoms in NC/Nga mice, we imaged skin lesions, evaluated dermatitis scores, and measured ear thickness. On day 21, the ear and dorsal skins of DNCB-treated NC/Nga mice showed severe erythema, erosion, and dryness (Fig. 2A). Co-treatment of 7,8,4′-THIF or tacrolimus, decreased AD-like symptom severity (Fig. 2A). The dermatitis score was gradually increased in DNCB-treated NC/Nga mice (Fig. 2B). On day 21, the dermatitis score was significantly higher in DNCB-treated NC/Nga mice (6.78±0.79) compared with naïve control (0.22±0.16) (Fig. 2B). Treatment with 200 nmol 7,8,4′-THIF (4.78±0.58), 400 nmol 7,8,4′-THIF (4.78±0.58), or tacrolimus (4.78±0.29) significantly decreased DNCB-induced increases in dermatitis score (Fig. 2B). Ear thickness was gradually increased in DNCB-treated NC/Nga mice (Fig. 2C). On day 21, the ears of DNCB-treated NC/Nga mice (0.50±0.02 mm) were significantly thicker than those of naïve control mice (0.21±0.00 mm) (Fig. 2C). Treatment with 200 nmol 7,8,4′-THIF (0.37±0.02 mm), 400 nmol 7,8,4′-THIF (0.39±0.02 mm), or tacrolimus (0.38±0.01 mm) markedly attenuated the DNCB-induced increase in ear thickness (Fig. 2C). On day 21, the ear skin of each mouse was prepared and examined. The ears of DNCB-treated NC/Nga mice became swollen and exhibited epidermal hypertrophy (Fig. 2D). In contrast, the ears of 7,8,4′-THIF or tacrolimus-treated mice showed less severe epidermal hypertrophy than DNCB-treated NC/Nga mice (Fig. 2D).

### 7,8,4′-THIF alleviated DNCB-induced scratching behavior in NC/Nga mice

**Figure 3:**
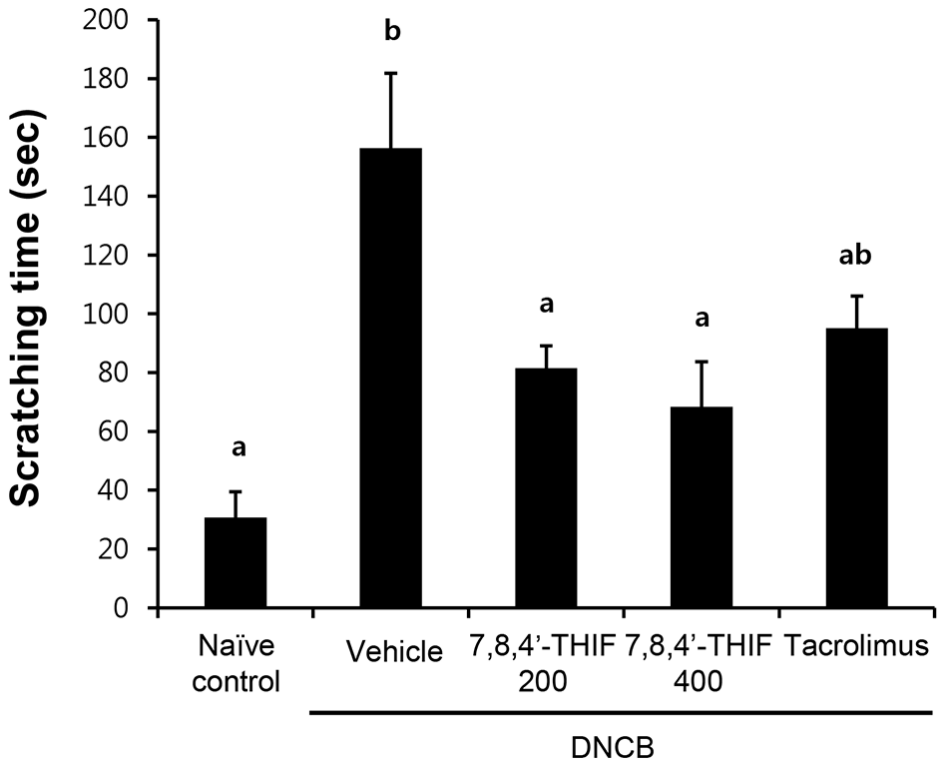
Effect of 7,8,4′-THIF on DNCB-induced scratching incidence in NC/Nga mice. Scratching time was evaluated on the last day of the experiment (day 21). Data represent the mean ± SEM (n = 9). Means with letters (a–b) within a graph are significantly different from each other at *p*<0.05.

Analysis of spontaneous scratching behavior in NC/Nga mice is a possible approach to evaluate the effectiveness of anti-pruritics [28]. To investigate the effect of 7,8,4′-THIF on DNCB-induced scratching behavior in NC/Nga mice, we monitored the animals and quantified the time they spent rubbing their nose, ears, and dorsal skin with their hind paws as scratching time. On day 21, scratching time was markedly increased in DNCB-treated NC/Nga mice (156.33±25.47 sec) compared with naïve control mice (30.69±8.84 sec), whereas treatment with 200 nmol 7,8,4′-THIF (81.46±7.61 sec), 400 nmol 7,8,4′-THIF (68.34±15.38 sec), or tacrolimus (95.13±10.93 sec) reduced the DNCB-increased scratching time (156.33±25.47 sec) (Fig. 3).

### 7,8,4′-THIF decreased DNCB-induced infiltration of eosinophils and mast cells into skin lesions in NC/Nga mice

**Figure 4:**
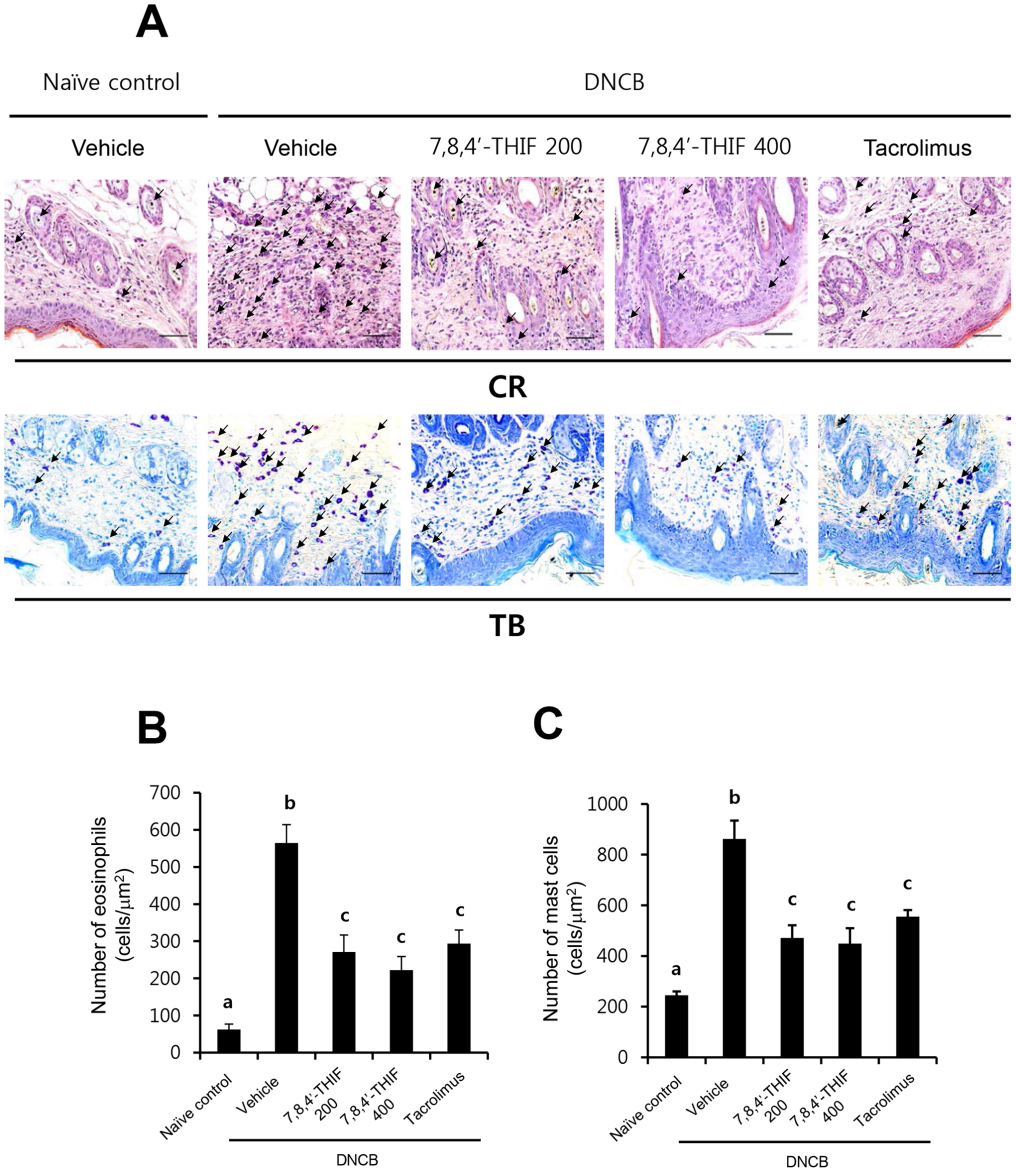
Effect of 7,8,4′-THIF on DNCB-induced eosinophil and mast cell infiltration in skin lesions of NC/Nga mice. (A) Representative images depicting the histological features of skin collected on day 21 are shown. Congo red (CR) and toluidine blue (TB) staining were used to identify eosinophils and mast cells, respectively. The arrows indicate the CR-stained eosinophils and the TB-stained mast cells. Cells were counted under a microscope at 400× magnification. The numbers of (B) eosinophils and (C) mast cells in 1 µm^2^ of skin were calculated. Data represent the mean ± SEM (n = 9). Means with letters (a–b) within a graph are significantly different from each other at *p*<0.05.

To investigate the effect of 7,8,4′-THIF on DNCB-induced infiltration of eosinophils and mast cells into skin lesions in NC/Nga mice, tissue sections collected on the last day of the experiment (day 21), were stained with CR and TB to identify eosinophils and mast cells, respectively. The number of CR-stained eosinophils per µm^2^ in skin lesions of DNCB-treated NC/Nga mice (564.44±49.64 cells) was significantly increased compared with that of naïve control mice (62.22±15.07 cells) (Fig. 4A and B). In contrast, topical application of 200 nmol 7,8,4′-THIF (271.11±45.60 cells), 400 nmol 7,8,4′-THIF (222.22±36.58 cells), or tacrolimus (293.33±37.11 cells) markedly lowered the number of eosinophils in the dorsal skin of DNCB-treated NC/Nga mice (Fig. 4A and B). Furthermore, the number of TB-stained mast cells per µm^2^ in skin lesions of DNCB-treated NC/Nga mice (862.22±72.45 cells) was significantly increased compared to that of naïve control mice (244.44±15.56 cells) (Fig. 4A and C). Treatment with 200 nmol 7,8,4′-THIF (471.11±49.79 cells), 400 nmol 7,8,4′-THIF (448.89±60.65 cells), or tacrolimus (555.56±25.34 cells) significantly reduced the number of infiltrated mast cells in the dorsal skin of DNCB-treated NC/Nga mice (Fig. 4A and C).

### 7,8,4′-THIF decreased the DNCB-induced serum IgE level increase in NC/Nga mice

**Figure 5:**
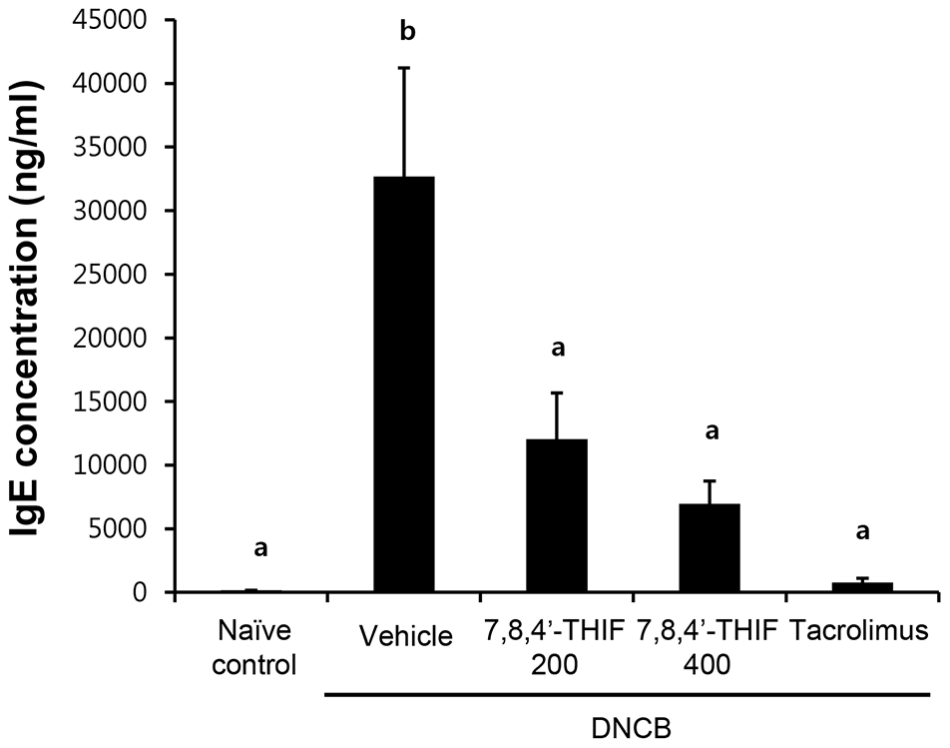
Effect of 7,8,4′-THIF on DNCB-induced increase in serum IgE level in NC/Nga mice. Blood was collected on the last day of the experiment (day 21). The level of serum IgE was measured with ELISA. Data are the means ± SEM (n = 6). Means with letters (a–c) within a graph are significantly different from each other at *p*<0.05.

To investigate the effect of 7,8,4′-THIF on DNCB-induced increase in serum IgE level in NC/Nga mice, blood samples were collected on the last day of the experiment (day 21). Repeated topical application of DNCB significantly increased serum IgE levels in DNCB-treated NC/Nga mice (32,695.3±8,513.29 ng/ml) compared to naïve control mice (155.25±10.61 ng/ml) (Fig. 5). However, treatment with 200 nmol 7,8,4′-THIF (12,055.2±3,620.46 ng/ml), 400 nmol 7,8,4′-THIF (6,947.13±1,808.97 ng/ml), or tacrolimus (767±331.45 ng/ml) significantly decreased the level of serum IgE in DNCB-treated NC/Nga mice (Fig. 5).

### 7,8,4′-THIF decreased DNCB-induced increase in TEWL in NC/Nga mice

**Figure 6:**
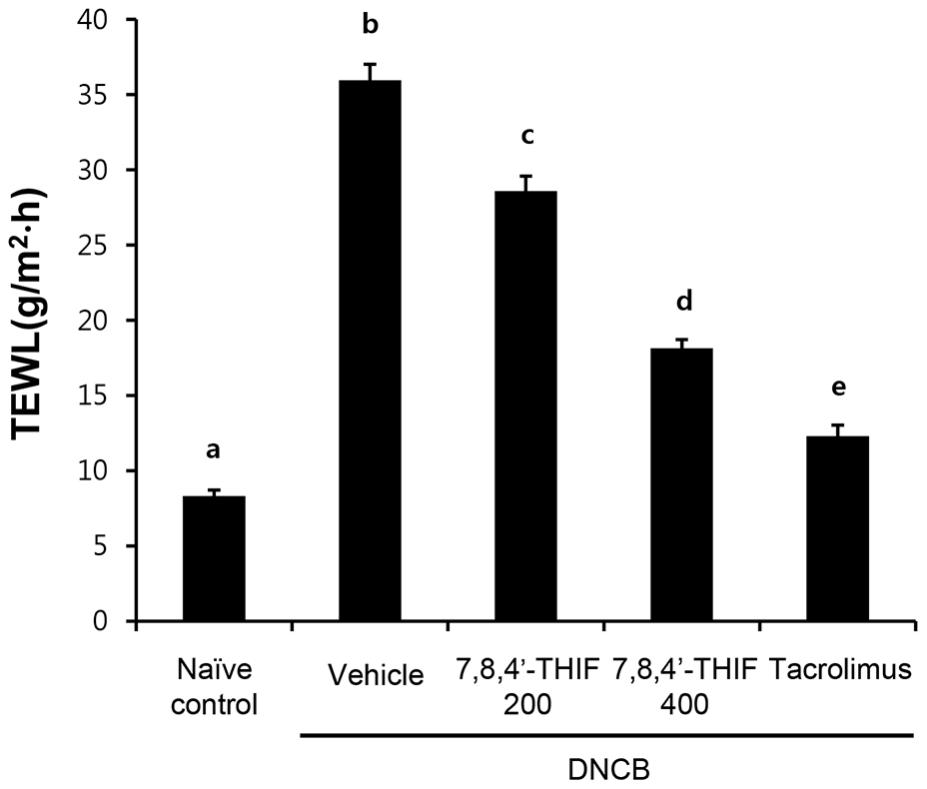
Effect of 7,8,4′-THIF on DNCB-induced increase in TEWL in NC/Nga mice. The level of TEWL in mouse dorsal skin was measured using a skin evaporative water recorder on the last day of the experiment (day 21). Data are the means ± SEM (n = 9). Means with letters (a–e) within a graph are significantly different from each other at *p*<0.05.

To investigate the effect of 7,8,4′-THIF on DNCB-induced loss of water through epidermal layer in NC/Nga mice, TEWL was measured on the last day of the experiment (day 21). DNCB-treated NC/Nga mice (35.96±1.06 g/m^2^⋅h) showed an increase in TEWL compared to naïve control mice (8.31±0.40 g/m^2^⋅h) (Fig. 6). Treatment with 200 nmol 7,8,4′-THIF (28.60±0.99 g/m^2^⋅h), 400 nmol 7,8,4′-THIF (18.14±0.58 g/m^2^⋅h), or tacrolimus (12.30±0.73 g/m^2^⋅h) significantly reduced TEWL in DNCB-treated NC/Nga mice (Fig. 6).

### 7,8,4′-THIF decreased DNCB-induced increases in TARC and Th2 and Th1 cytokines in NC/Nga mice

We examined chemokine and cytokine levels in dorsal skins collected on the last day of the experiment (day 21). We found that the chemokine TARC was markedly increased in skin lesions in DNCB-treated NC/Nga mice compared to naïve control mice (Table 1). However, treatment with 200 nmol 7,8,4′-THIF, 400 nmol 7,8,4′-THIF, or tacrolimus significantly reduced TARC levels in dorsal skins of DNCB-treated NC/Nga mice (Table 1).

**Table 1:** Effects of 7,8,4′-THIF on DNCB-induced increase in chemokine TARC and Th2 and Th1 cytokines in NC/Nga mice.

Th2 chemokine	TARC
Naïve control+vehicle	99.70±7.06^a^
DNCB+vehicle	133.96±8.00^b^
DNCB+7,8,4′-THIF 200	103.80±3.45^a^
DNCB+7,8,4′-THIF 400	87.05±2.86^a^
DNCB+tacrolimus	108.82±4.36^a^

Th2 cytokines	IL-4	IL-5	IL-13
Naïve control+vehicle	102.58±5.25^a^	25.63±1.41^a^	378.31±9.26^a^
DNCB+vehicle	157.10±17.31^b^	47.43±4.48^b^	560.88±32.79^b^
DNCB+7,8,4′-THIF 200	100.77±8.77^a^	31.08±2.45^a^	368.14±18.28^a^
DNCB+7,8,4′-THIF 400	85.93±3.86^a^	31.81±2.68^a^	331.23±9.15^a^
DNCB+tacrolimus	83.23±6.47^a^	30.97±2.89^a^	293.75±14.05^a^

Th1 cytokines	IL-12	IFN-γ
Naïve control+vehicle	95.53±4.25^a^	94.24±4.43^a^
DNCB+vehicle	117.85±11.82^b^	148.04±13.19^b^
DNCB+7,8,4′-THIF 200	85.34±9.84^ac^	101.58±8.96^a^
DNCB+7,8,4′-THIF 400	62.66±3.64^a^	86.12±5.83^a^
DNCB+tacrolimus	62.41±4.59^c^	83.15±9.10^a^

The pg/ml levels of Th-derived chemokine TARC and cytokines in NC/Nga mice are shown. Dorsal skins were collected on the last day of the experiment (day 21). The levels of TARC; Th2 cytokines IL-4, IL-5, and IL-13; and Th1 cytokines IL-12 and IFN-γ in dorsal skin were measured with ELISAs. Data are the means ± SEM (n = 6). Means with letters (a–c) are significantly different from each other at *p*<0.05.

On the other hand, the levels of Th2 cytokines IL-4, IL-5, and IL-13 were significantly increased in skin lesions of DNCB-treated NC/Nga mice compared with naïve control mice (Table 1). The levels of Th1 cytokines IL-12 and IFN-γ were also significantly increased in DNCB-treated NC/Nga mice skin lesions compared with naïve control mice (Table 1). However, treatment with 200 nmol 7,8,4′-THIF, 400 nmol 7,8,4′-THIF, or tacrolimus significantly decreased the level of Th2 and Th1 cytokines IL-4, IL-5, IL-13, IL-12, and IFN-γ in the dorsal skin of DNCB-treated NC/Nga mice (Table 1).

## Discussion

Our results demonstrate that topical application of 7,8,4′-THIF attenuated DNCB-induced AD-like symptoms in NC/Nga mice. 7,8,4′-THIF treatment significantly alleviated DNCB-induced increases in skin lesion severity, dermatitis score, ear thickness, scratching behavior, and serum IgE levels. In addition, DNCB-induced infiltration of skin lesions by eosinophils and mast cells was decreased following 7,8,4′-THIF treatment. In addition, we observed that 7,8,4′-THIF recovered epidermal water loss in DNCB-treated NC/Nga mice. We also found that 7,8,4′-THIF treatment lowered levels of the chemokine TARC; Th2 cytokines IL-4, IL-5, and IL-13; and Th1 cytokines IL-12 and IFN-γ in DNCB-treated NC/Nga mice. These findings suggest that topical application of 7,8,4′-THIF might be useful for the treatment of AD.

Various experimental and clinical investigations have reported that natural immune modulators from herbal extracts or derivatives may have therapeutic effects on AD [29]–[31]. Epidemiological studies suggest that soy isoflavones have beneficial effects on health, including antioxidant activity, cancer prevention, and enhanced immunity [32]–[34]. The main soy isoflavone glucosides daidzin and genistin are hydrolyzed by intestinal microorganisms to the aglycones daidzein and genistein, respectively, prior to absorption [35]. Daidzin and its metabolites have been shown to have cancer preventive effects [36], [37]; however, there have been no reports of their possible anti-AD effects.

Our results indicated that 7,8,4′-THIF, a metabolite of the soy isoflavone daidzin, suppresses the development of DNCB-induced dermatitis in NC/Nga mice, probably by down-regulating various Th2- (TARC, IL-4, IL-5, and IL-13) and Th1-associated factors (IL-12 and IFN-γ). The biological consequences of such changes in this model include highly inhibited epidermal and dermal thickness, decreased infiltration of eosinophils and mast cells, and lower serum IgE production. Of particular interest is a decrease in the levels of Th2-associated factors, TARC, IL-4, IL-5, and IL-13. TARC produced by Th2 cells and keratinocytes is thought to attract Th2 cells and induce the pathological responses typically associated with AD [7], [8]. IL-4 and IL-13 induce isotype switching to IgE synthesis by B cells, which activates mast cells [11], [38]. IL-5 is known to be a potent chemoattractant for eosinophils and plays an important role in eosinophil development and survival [11]. The Th1-associated factors IL-12 and IFN-γ are associated with AD severity [39], [40]. Decrease in IL-12 and IFN-γ levels might play a role in the effects of 7,8,4′-THIF in mice. Recent *in vitro* observations have demonstrated that eosinophils synthesize biologically active IL-12, an activity that can be strongly induced by Th2-type cytokines, including IL-4 [39]. Cytokines derived from Th2-type cells that present in the early phase of atopic eczema may attract eosinophils, stimulate IL-12 expression, and thereby cause sequential activation of Th0- and Th1-type cells [39]. IFN-γ produced by Th1 cells activates keratinocytes to express Fas (CD95), which predisposes keratinocytes to apoptosis, leading to the formation of eczematous lesions [40], [41].

The epidermis provides an important physical barrier against the environment. AD is characterized by a defective skin barrier that allows increased allergen and pathogen penetration [3], [42]–[44]. TEWL is linked to damage of the stratum corneum lipid barrier and a subsequent loss of corneocyte adhesion [45]. Skin barrier alterations that cause increased TEWL and decreased skin hydration underlie severity and itch intensity in AD [3], [45]. Recent studies have demonstrated that TEWL correlates well with AD disease activity [46]. In our study, TEWL was increased in DNCB-treated NC/Nga mice compared to naïve control mice. Elevated TEWL was reduced in 7,8,4′-THIF treated mice, suggesting that 7,8,4′-THIF may help to maintain epidermal skin barrier function and modulate AD disease severity.

Although the detailed molecular mechanism(s) underlying the therapeutic effects of 7,8,4′-THIF remains to be elucidated, our results clearly show that topical 7,8,4′-THIF application alleviates AD-like skin lesions by modifying both local and systemic inflammation. These effects were demonstrated by changes in ear thickness and decreased infiltration of inflammatory cells, TEWL, chemokine TARC, Th2 and Th1 cytokines, and IgE. Given the fact that AD prevalence is steadily increasing in all major developed countries and that there is virtually no effective treatment, our findings in DNCB-treated NC/Nga mice suggest that 7,8,4′-THIF may be an effective therapeutic agent that can be used to attenuate the development of AD.
